# Efficacy and safety of direct-acting oral anticoagulants compared to vitamin K antagonists in COVID-19 outpatients with cardiometabolic diseases

**DOI:** 10.1186/s12933-021-01368-6

**Published:** 2021-09-04

**Authors:** José Miguel Rivera-Caravaca, Stephanie L. Harrison, Benjamin J. R. Buckley, Elnara Fazio-Eynullayeva, Paula Underhill, Francisco Marín, Gregory Y. H. Lip

**Affiliations:** 1 Liverpool Centre for Cardiovascular Science, University of Liverpool and Liverpool Heart & Chest Hospital, Liverpool, UK; 2 Department of Cardiology, Hospital Clínico Universitario Virgen de La Arrixaca, University of Murcia, Instituto Murciano de Investigación Biosanitaria (IMIB-Arrixaca), CIBERCV, Murcia, Spain; 3 Liverpool University Hospitals NHS Foundation Trust, Liverpool, UK; 4 Cardiovascular and Metabolic Medicine, Institute of Life Course and Medical Sciences, University of Liverpool, Liverpool, UK; 5 TriNetX LLC., Cambridge, MA USA; 6 TriNetX LLC., London, UK; 7 Department of Clinical Medicine, Aalborg University, Aalborg, Denmark

**Keywords:** Coronavirus disease 2019, SARS-CoV-2, Thrombosis, Anticoagulant, Vitamin K antagonist, Direct-acting oral anticoagulants, Bleeding

## Abstract

**Background:**

It remains uncertain if prior use of oral anticoagulants (OACs) in COVID-19 outpatients with multimorbidity impacts prognosis, especially if cardiometabolic diseases are present. Clinical outcomes 30-days after COVID-19 diagnosis were compared between outpatients with cardiometabolic disease receiving vitamin K antagonist (VKA) or direct-acting OAC (DOAC) therapy at time of COVID-19 diagnosis.

**Methods:**

A study was conducted using TriNetX, a global federated health research network. Adult outpatients with cardiometabolic disease (i.e. diabetes mellitus and any disease of the circulatory system) treated with VKAs or DOACs at time of COVID-19 diagnosis between 20-Jan-2020 and 15-Feb-2021 were included. Propensity score matching (PSM) was used to balance cohorts receiving VKAs and DOACs. The primary outcomes were all-cause mortality, intensive care unit (ICU) admission/mechanical ventilation (MV) necessity, intracranial haemorrhage (ICH)/gastrointestinal bleeding, and the composite of any arterial or venous thrombotic event(s) at 30-days after COVID-19 diagnosis.

**Results:**

2275 patients were included. After PSM, 1270 patients remained in the study (635 on VKAs; 635 on DOACs). VKA-treated patients had similar risks and 30-day event-free survival than patients on DOACs regarding all-cause mortality, ICU admission/MV necessity, and ICH/gastrointestinal bleeding. The risk of any arterial or venous thrombotic event was 43% higher in the VKA cohort (hazard ratio 1.43, 95% confidence interval 1.03–1.98; Log-Rank test p = 0.029).

**Conclusion:**

In COVID-19 outpatients with cardiometabolic diseases, prior use of DOAC therapy compared to VKA therapy at the time of COVID-19 diagnosis demonstrated lower risk of arterial or venous thrombotic outcomes, without increasing the risk of bleeding.

**Supplementary Information:**

The online version contains supplementary material available at 10.1186/s12933-021-01368-6.

## Introduction

Oral anticoagulants (OACs), including vitamin K antagonists (VKAs) and direct-acting OACs (DOACs), have been used for thromboprophylaxis in different clinical scenarios. In the pivotal clinical trials of stroke prevention in atrial fibrillation (AF), DOACs were non-inferior to warfarin for preventing stroke/systemic embolism (SE), with lower rates of intracranial haemorrhage (ICH) in comparison with warfarin [1–4]. Similarly, in venous thromboembolism (VTE), dabigatran, rivaroxaban, apixaban and edoxaban were non-inferior to conventional therapy in terms of efficacy and caused less bleeding in a broad spectrum of patients [5–9]. These trials evidences are supported by data from real world and observational studies, where DOACs have demonstrated significantly lower rates for major bleeding and a positive net clinical benefit compared to VKAs [10–13]. In VTE patients, the use of DOACs has also been associated with a lower risk of VTE recurrence even after anticoagulant discontinuation [14, 15].

Coronavirus Disease 2019 (COVID-19) has shown to trigger endothelial dysfunction, inflammatory and hypercoagulable states [16–18]. The risk of thrombosis is increased, and thromboembolic complications are relatively frequent in these patients, particularly in those patients with intensive care unit (ICU) admission [19–22]. Thus, anticoagulation is now well-established for the management of COVID-19 patients [23–25]. However, a common limitation is that most of the evidence to date refers to the hospitalization context. Thus, it remains uncertain if prior OAC therapy in outpatients, especially amongst patients with multimorbidity, would potentially influence the severity and clinical outcomes after COVID-19 diagnosis.

The aim of this study was to compare clinical outcomes 30-days after COVID-19 diagnosis between outpatients with cardiometabolic disease on chronic VKA or DOAC therapy at time of COVID-19 diagnosis, using a propensity score matching (PSM) approach.

## Methods

Data from TriNetX, a global federated health research network with real-time updates of anonymised electronic medical records (EMRs) mainly from the United States (US), were used. The network includes healthcare organisations (HCOs, academic medical centres, specialty physician practices and community hospitals), with accumulated data for more than 71 million patients. Approximately, 18 million adult patients had a visit in a TriNetX HCO during 2020.

The inclusion criteria were ≥ 18 years and outpatient with COVID-19 and cardiometabolic disease recorded in EMRs between 20 January 2020 and 15 February 2021. COVID-19 was identified using criteria provided by TriNetX based on Centers for Disease Control and Prevention (CDC) coding guidelines [26]. COVID-19 status was determined using codes in EMRs or a positive test result identified with COVID-19-specific laboratory codes. Specifically, COVID-19 was identified by one or more of the International Classification of Diseases, Tenth Revision, Clinical Modification (ICD-10-CM) codes in the EMRs of the patients (Additional file 1: Table S1). The inclusion date start was set as 20 January 2020 because COVID-19 was first confirmed in the US on this date, and the TriNetX network is predominately US-based [27]. Cardiometabolic disease was defined as the combination of diabetes mellitus (ICD-10-CM code: E08-E13) and any disease of the circulatory system (ICD-10-CM code: I00-I99). In addition, all patients should have OAC therapy in the one-year period prior to COVID-19 recorded in their EMRs, and remained on this therapy at COVID-19 diagnosis. During 1-year period prior to COVID-19 diagnosis, patients must not be hospitalized to ensure they are stable outpatients.

All patients were then stratified by OAC prescription. The DOAC group included outpatients who received either dabigatran, apixaban, rivaroxaban or edoxaban for at least 1 year before COVID-19 diagnosis, whereas the VKA group included outpatients who received warfarin under the same conditions. Patients were excluded if they received concomitant anticoagulant therapy (oral or parenteral). Baseline demographics, comorbidities and medication use were also captured from the patient EMRs.

The searches were run in TriNetX on 30 April 2021, which allowed for at least 30-days of follow-up for all participants from the time all conditions were fulfilled. When the searchers were run, there were 61 participating HCOs within the TriNetX research network.

### Follow-up and clinical outcomes

All patients were followed-up for up to 30-days after COVID-19 diagnosis. Primary outcomes included all-cause mortality, ICU admission/mechanical ventilation (MV) necessity, ICH/gastrointestinal bleeding, and the composite of any arterial or venous thrombotic event (any of the following: myocardial infarction, other arterial thrombosis, VTE, or ischemic stroke/transient ischemic attack [TIA]/SE). The secondary outcomes were hospital admission, myocardial infarction, VTE, ischemic stroke/TIA/SE, and all bleeding. Further details about the ICD-10-CM codes used for the identification of every outcome are included in Additional file 1: Table S2.

### Ethical issues

As a federated network, research studies using TriNetX do not require ethical approval. To comply with legal frameworks and ethical guidelines guarding against data re-identification, the identity of participating HCOs and their individual contribution to each dataset are not disclosed. The TriNetX platform only uses aggregated counts and statistical summaries of de-identified information. No protected health information or personal data are made available to the users of the platform.

### Statistical analysis

Continuous variables were expressed as mean and standard deviation (SD), and tested for differences with independent-sample *t* tests. Categorical variables were expressed as absolute frequencies and percentages, and tested for differences with chi-squared test.

The TriNetX platform was used to run 1:1 PSM using logistic regression. The platform uses ‘greedy nearest-neighbour matching’ with a caliper of 0.1 pooled standard deviations and difference between propensity scores ≤ 0.1. We assessed covariate balance between groups using standardised mean differences (SMDs). Any baseline characteristic with a SMD between cohorts lower than 0.1 is considered well matched [28].

Cox proportional Hazard Ratios (HRs) with 95% confidence intervals (CI) for 30-days outcomes were calculated following PSM. Kaplan–Meier survival curves were also produced with Log-Rank tests after PSM. No imputations were made for missing data. Two-sided p-values < 0.05 were accepted as statistically significant. Statistical analysis was performed using the TriNetX Analytics function in the online research platform.

## Results

Overall, 2275 patients (mean age 67.7 ± 12.8 years, 1222 [53.7%] males) with COVID-19 and cardiometabolic disease were included. Of these, 648 (363 [56.0%] males, mean age of 67.9 ± 12.9 years) were on VKA therapy at the time of COVID-19 diagnosis, and 1627 (859 [52.8%] males, mean age 67.6 ± 12.8 years) were on DOACs.

There were no differences between cohorts regarding the main reasons for OAC (i.e. AF or previous pulmonary embolism). Other VTEs were more common in patients taking VKA therapy. In addition, patients on VKA were in general more comorbid, as demonstrated by the higher prevalence of hypertension, heart failure, ischemic heart disease, hyperlipidaemia, overweight/obesity, diseases of the nervous and digestive systems, acute kidney failure/chronic kidney disease, and neoplasms (Table 1). After PSM, 1270 remained in the study, 635 individuals on VKA therapy and 635 on DOACs (i.e. proportion of 1:1), well balanced on age, gender, ethnicity, and comorbidities (Table 1).

**Table 1:** Comparison of clinical characteristics of the study cohort before and after propensity score matching

	Initial populations						Propensity score matched populations					
	COVID-19 patients on prior VKA N = 648		COVID-19 patients on prior DOAC N = 1627		p-value	SMD	COVID-19 patients on prior VKA N = 635		COVID-19 patients on prior DOAC N = 635		p-value	SMD
Age (years), mean (SD)	67.90	12.88	67.61	12.79	0.636	0.022	67.80	12.90	68.29	12.26	0.492	0.039
Male sex	363	56.02%	859	52.80%	0.164	0.065	353	55.59%	371	58.43%	0.308	0.057
Ethnicity												
Not Hispanic or Latino	494	76.24%	1133	69.64%	0.002	0.149	482	75.91%	481	75.75%	0.948	0.004
Hispanic or Latino	65	10.03%	117	7.19%	0.024	0.101	64	10.08%	59	9.29%	0.635	0.027
Unknown Ethnicity	89	13.74%	377	23.17%	0.481	0.245	89	14.02%	95	14.96%	0.632	0.027
Comorbidities												
Diabetes mellitus	648	100%	1627	100%	1.000	0.000	635	100%	635	100%	1.000	< 0.001
Hypertension	613	94.60%	1479	90.90%	0.003	0.143	600	94.49%	602	94.80%	0.803	0.014
Heart failure	270	41.67%	574	35.28%	0.004	0.132	265	41.73%	249	39.21%	0.360	0.051
Ischemic heart disease	322	49.69%	721	44.32%	0.020	0.108	314	49.45%	311	48.98%	0.866	0.009
Atrial fibrillation	393	60.65%	984	60.48%	0.941	0.003	386	60.79%	389	61.26%	0.863	0.010
Peripheral vascular disease	105	16.20%	239	14.69%	0.363	0.042	103	16.22%	96	15.12%	0.589	0.030
Hyperlipidemia	570	87.96%	1322	81.25%	< 0.001	0.187	557	87.72%	553	87.09%	0.735	0.019
Cerebrovascular disease	170	26.24%	367	22.56%	0.062	0.086	166	26.14%	160	25.20%	0.700	0.022
Cerebral infarction	81	12.50%	176	10.82%	0.253	0.052	81	12.76%	85	13.39%	0.739	0.019
Pulmonary embolism	124	19.14%	266	16.35%	0.111	0.073	121	19.06%	121	19.06%	1.000	0.000
Other venous embolism and thrombosis	195	30.09%	391	24.03%	0.003	0.137	187	29.45%	189	29.76%	0.902	0.007
Overweight/obesity	397	61.27%	913	56.12%	0.025	0.105	387	60.94%	389	61.26%	0.908	0.006
Diseases of the respiratory system	543	83.80%	1307	80.33%	0.056	0.090	532	83.78%	523	82.36%	0.501	0.038
Diseases of the nervous system	528	81.48%	1264	77.69%	0.046	0.094	517	81.42%	518	81.58%	0.942	0.004
Diseases of the digestive system	526	81.17%	1230	75.60%	0.004	0.136	514	80.94%	508	80.00%	0.671	0.024
Acute kidney failure and chronic kidney disease	287	44.29%	582	35.77%	< 0.001	0.175	277	43.62%	272	42.84%	0.777	0.016
Diseases of liver	130	20.06%	290	17.82%	0.214	0.057	129	20.32%	136	21.42%	0.629	0.027
Neoplasms	351	54.17%	766	47.08%	0.002	0.142	341	53.70%	345	54.33%	0.822	0.013
Pharmacological therapy												
Beta blockers	495	76.39%	1228	75.48%	0.647	0.021	485	76.38%	485	76.38%	1.000	< 0.001
ACE inhibitors	396	61.11%	836	51.38%	< 0.001	0.197	385	60.63%	385	60.63%	1.000	< 0.001
Angiotensin II inhibitors	237	36.57%	621	38.17%	0.479	0.033	236	37.17%	231	36.38%	0.771	0.016
Alpha blockers	549	84.72%	1282	78.80%	0.001	0.154	537	84.57%	542	85.35%	0.695	0.022
Antilipemic agents	366	56.48%	863	53.04%	0.137	0.069	359	56.54%	349	54.96%	0.572	0.032
Calcium channel blockers	477	73.61%	1091	67.06%	0.002	0.144	466	73.39%	463	72.91%	0.849	0.011
Diuretics	455	70.22%	1065	65.46%	0.030	0.102	445	70.08%	438	68.98%	0.670	0.024
Antiarrhythmics	401	61.88%	988	60.73%	0.609	0.024	396	62.36%	387	60.95%	0.603	0.029
Antiplatelets	495	76.39%	1228	75.48%	0.647	0.021	485	76.38%	485	76.38%	1.000	< 0.001
Blood glucose regulation agents (including oral antidiabetics and insulin)	554	85.50%	1389	85.37%	0.947	0.003	545	85.83%	545	85.83%	1.000	< 0.001

ACE: Angiotensin-converting enzyme; DOAC: direct-acting oral anticoagulant; SD: standard deviation; SMD: standardized mean difference; VKA: vitamin K antagonist

### Comparisons of clinical outcomes

In the initial populations, all event rates were numerically higher in the COVID-19 patients with cardiometabolic disease on prior VKA, which were significant for any arterial or venous thrombotic event (14.04% vs. 8.54%, p < 0.001), VTE (9.88% vs. 6.82%, p = 0.007), and ischemic stroke/TIA/SE (4.17% vs. 1.72%, p < 0.001). After PSM, the rate of any arterial or venous thrombotic event (14.02% vs. 9.61%, p = 0.015) and ischemic stroke/TIA/SE (4.25% vs. 1.73%, p = 0.008) remained higher in users of VKA compared to users of DOACs. Further details are shown in Table 2.

**Table 2:** Primary and secondary outcomes in patients on VKAs or DOAC at COVID-19 diagnosis, before (unmatched) and after propensity score matching

Outcomes	Initial populations			Propensity score matched populations		
	COVID-19 patients on prior VKA (N = 648)	COVID-19 patients on prior DOAC (N = 1627)	p-value	COVID-19 patients on prior VKA (N = 635)	COVID-19 patients on prior DOAC (N = 635)	p-value
All-cause mortality	10 (1.54%)	22 (1.35%)	0.913	10 (1.57%)	11 (1.73%)	0.826
ICU admission/MV necessity	12 (1.85%)	21 (1.29%)	0.378	12 (1.89%)	10 (1.57%)	0.667
ICH/gastrointestinal bleeding	10 (1.54%)	12 (0.74%)	0.145	10 (1.57%)	10 (1.57%)	1.000
Any arterial or venous thrombotic event	91 (14.04%)	139 (8.54%)	< 0.001	89 (14.02%)	61 (9.61%)	0.015
Hospital admission	17 (2.62%)	42 (2.58%)	0.891	17 (2.68%)	17 (2.68%)	1.000
Myocardial infarction	10 (1.54%)	13 (0.80%)	0.190	10 (1.57%)	10 (1.57%)	1.000
Venous thromboembolism	64 (9.88%)	111 (6.82%)	0.007	62 (9.76%)	50 (7.87%)	0.235
All bleeding events	16 (2.45%)	18 (1.11%)	0.084	16 (2.52%)	10 (1.57%)	0.235
Ischemic stroke/TIA/SE	27 (4.17%)	28 (1.72%)	< 0.001	27 (4.25%)	11 (1.73%)	0.008

DOAC: direct-acting oral anticoagulant; ICH: intracranial haemorrhage; ICU: intensive care unit; MV: mechanical ventilation; SE: systemic embolism; TIA: transient ischemic attack; VKA: vitamin K antagonist

**Fig. 1:**
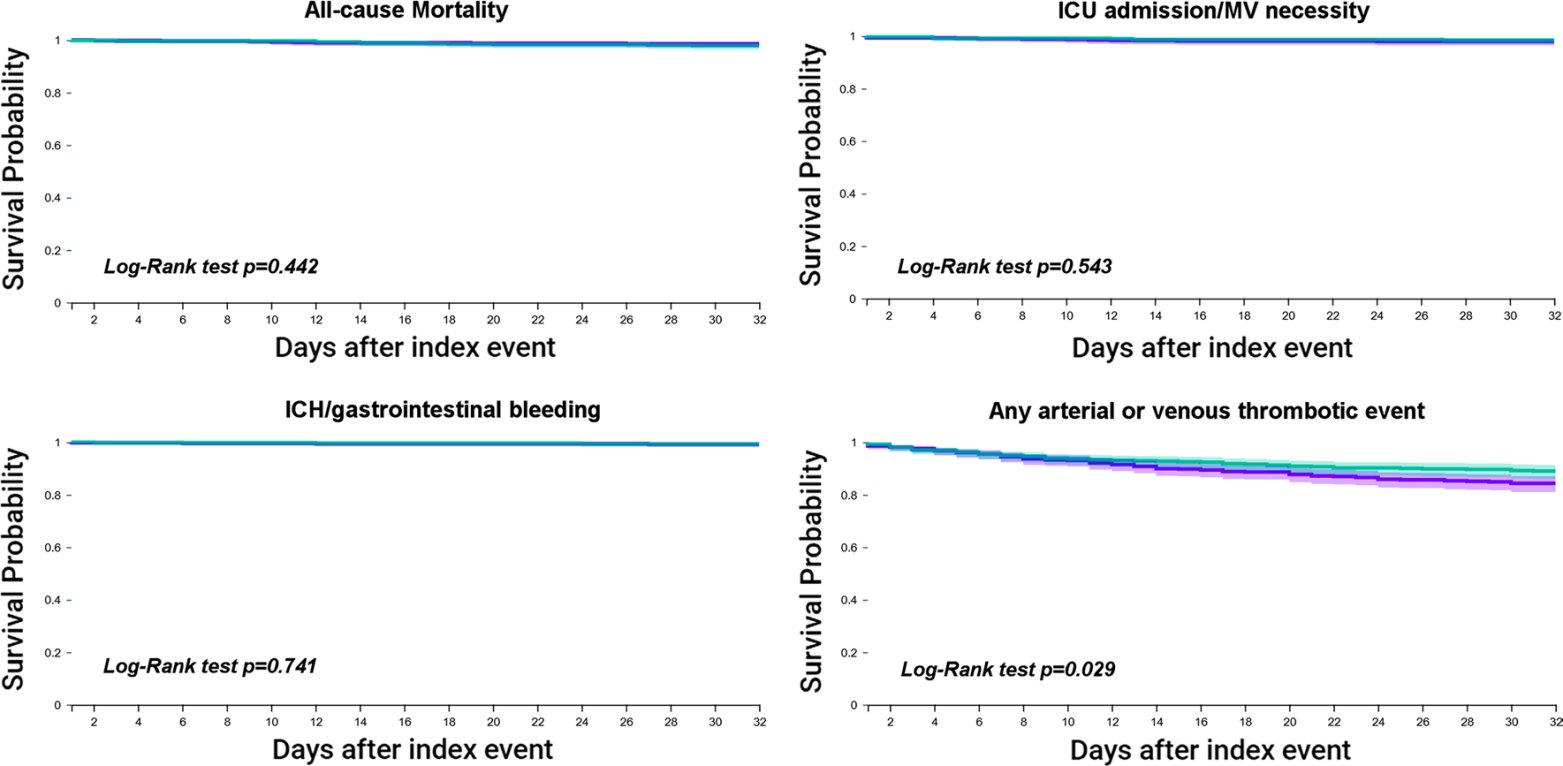
Comparison of survival curves for the primary outcomes between patients on VKAs or DOACs at COVID-19 diagnosis after propensity score matching. Purple line: Prior VKA use; Green line: Prior DOAC use

In terms of the primary outcomes after PSM, there were no significant differences in the risks of all-cause mortality, ICU admission/MV necessity, or ICH/gastrointestinal bleeding between patients on VKA or DOACs. Thus, the risk for all-cause mortality was similar comparing the VKA cohort and DOAC cohort (HR 0.70, 95% 0.28–1.74). The risk of ICU admission/MV necessity in the VKA cohort was not significantly different compared to the DOAC cohort (HR 1.31, 95% CI 0.55–3.10), and the risk of ICH/gastrointestinal bleeding was similar (HR 1.29, 95% CI 0.29–5.75). Event-free survival for these three outcomes was not different between cohorts, as assessed by the Kaplan–Meier analyses (Log-Rank tests: p = 0.442 for mortality; p = 0.543 for ICU admission/MV necessity; and p = 0.741 for ICH/gastrointestinal bleeding) (Fig. 1).

The composite outcome of any arterial or venous thrombotic event was inferior in the DOAC cohort in comparison to the VKA cohort, as demonstrated by the 43% higher risk in VKA users (HR 1.43, 95% CI 1.03–1.98) and the lower event-free survival (Log-Rank test p = 0.029) (Fig. 1).

### Secondary outcomes

**Fig. 2:**
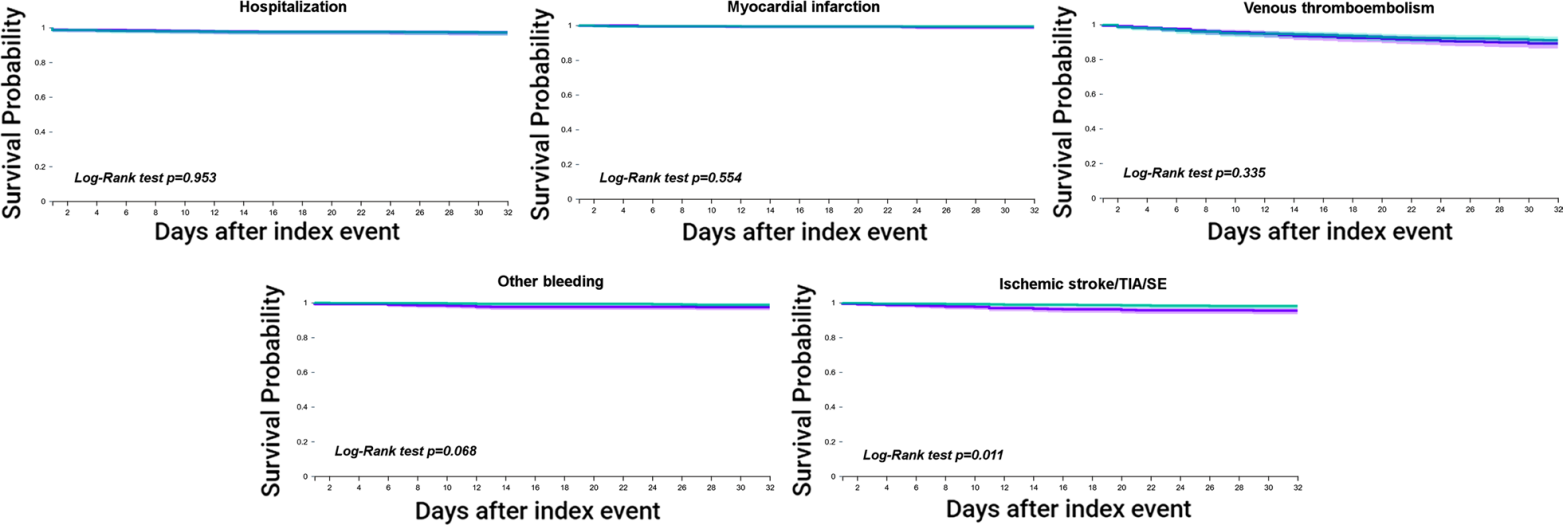
Comparison of survival curves for the secondary outcomes between patients on VKAs or DOACs at COVID-19 diagnosis after propensity score matching. Purple line: Prior VKA use; Green line: Prior DOAC use

Hospital admission was similar in VKA and DOAC patients (HR 0.98, 95% CI 0.50–1.92). Likewise, there were no significant differences in the risk of myocardial infarction between both groups (HR 1.46, 95% CI 0.41–5.18), nor in the risk of VTE (HR 1.20, 95% CI 0.83–1.74). The risk of all bleeding events was not significantly different in patients previously taking VKA (HR 2.24, 95% CI 0.92–5.44) (Fig. 2).

The risk of suffering an ischemic stroke/TIA/SE at 30-days after COVID-19 diagnosis was 2.42-fold higher in users of VKA compared to DOAC users (HR 2.42, 95% CI 1.20–4.88; Log-Rank test p = 0.011) (Fig. 2).

### Sensitivity analysis

A sensitivity analysis was performed including only those patients fulfilling the initial inclusion/exclusion criteria and AF, as this is the most common indication for OAC use. After PSM on a 1:1 proportion, 866 patients (mean age 71.2 ± 11.6 years, 508 [61%] males) with COVID-19, AF and other cardiometabolic disease were included, of which 433 (254 [58.7%] males, mean age of 71.1 ± 12.2 years) were on VKA therapy at the time of COVID-19 diagnosis, and 433 (254 [58.7%] males, mean age 71.3 ± 11.0 years) were on DOACs.

Overall, the risk for the primary outcomes in this sensitivity analysis between patients on VKA or DOACs was similar compared to the main analysis. Thus, there were no significant differences in VKA or DOAC-treated patients regarding all-cause mortality (HR 1.36, 95% CI 0.47–3.91; Log-Rank test p = 0.570), ICU admission/MV necessity (HR 1.35, 95% CI 0.57–3.19; Log-Rank test p = 0.499), and ICH/ gastrointestinal bleeding (HR 0.34, 95% CI 0.07–1.67; Log-Rank test p = 0.162). The risk of composite outcome of any arterial or venous thrombotic event was higher in the VKA cohort compared to the DOAC cohort (HR 1.80, 95% CI 1.04–3.12), with a significantly lower event-free survival (Log-Rank test p = 0.033).

Concerning the secondary outcomes, the risks of hospital admission (HR 0.94, 95% CI 0.46–1.90; Log-Rank test p = 0.859), myocardial infarction (OR 0.58, 95% CI 0.17–1.97; Log-Rank test p = 0.372), VTE (HR 1.48, 95% CI 0.69–3.19; Log-Rank test p = 0.315), and all bleeding events (HR 1.14, 95% CI 0.44–2.96; Log-Rank test p = 0.782), was also similar in patients previously taking VKA or DOACs. The risk of ischemic stroke/TIA/SE was increased in VKA users (HR 3.69, 95% CI 1.37–9.93), and Kaplan–Meier analysis demonstrated a significantly lower event-free survival in the VKA-treated cohort (Log-Rank test p = 0.006).

## Discussion

In this study including COVID-19 *outpatients* with cardiometabolic disease, patients taking VKA before COVID-19 diagnosis showed a 43% higher 30-day risk of any arterial/venous thrombotic event and ischemic stroke/TIA/SE, compared to patients taking DOAC, adjusting for comorbidities using PSM. These results were consistent also in COVID-19 patients with AF and other cardiometabolic disease.

Previous studies have shown that cardiometabolic multimorbidity is common among patients with COVID-19, and it is associated with a higher risk of hospitalization and a worsened prognosis [29–32]. In particular, diabetes is frequent in these patients [33, 34], and associated with an increased risk of complications, likely due to clustering with other conditions [29]. Simultaneously, several cardiovascular diseases, including hypertension, cerebrovascular disease and coronary artery disease, are prevalent in COVID-19 patients, and are associated with a higher risk of adverse outcomes [35]. Given that the presence of cardiometabolic disease is not uncommon in patients with Severe Acute Respiratory Syndrome Coronavirus 2 (SARS-CoV-2) infection, this might have major implications for prognosis in this condition.

It is well established that COVID-19 increases the risk of arterial and venous thrombosis [20, 21], leading to the research focus on thromboinflammation and antithrombotic therapy, particularly in anticoagulation therapy [23, 25, 36, 37]. Many patients had preexisting cardiovascular diseases and were already on OAC therapy when they were diagnosed of COVID-19 [38]. Hence, the role of prior OAC therapy in the context of COVID-19 is gaining interest. For example, a recent study concluded that prior use of therapeutic anticoagulation was not associated with improved survival in hospitalized COVID-19 patients [39]. Similarly, a small study found that regular VKA use in hospitalized frail older patients with COVID-19 was associated with increased mortality during the first week [40]. On the contrary, a retrospective study concluded that COVID-19 patients on OAC at the time of infection and throughout their disease course had significantly lower risk of all-cause mortality at 21 days [41]. Indeed, OAC therapy was associated with lower risk of all-cause mortality in elderly AF patients with COVID-19 [42], and more recently, the ACTION trial showed that among patients admitted with COVID-19 and elevated D-dimer, therapeutic anticoagulation was not superior to prophylactic anticoagulation; and rivaroxaban for stable patients and enoxaparin for unstable patients increased major bleeding without improving clinical outcomes [43, 44].

Nevertheless, a common limitation of most studies (both, those with positive and negative results in favour of OACs) is the hospitalization setting. Such patients have already suffered deterioration of their baseline status, which has led to hospitalization and may be a manifestation of a more severe state of the SARS-CoV-2 infection, with an increased risk of thrombosis, mortality, ICU admission, and MV. In these more severe patients, several confounding factors may be acting when evaluating the potential role of previous OAC, and the differences between VKA and DOAC may be non-existent.

As a result, COVID-19 *outpatients* with cardiometabolic diseases under anticoagulation therapy are a population with scarce data, and their management could be particularly complex. A recent study concluded that OAC therapy in high-risk AF patients was associated with a lower risk of receiving a positive COVID-19 test and severe COVID-19 outcomes [45]. In turn, pre-admission and in-hospital OAC therapy (either VKA or DOAC) were positively associated with higher survival in a study including elderly AF patients with COVID-19 [46]. Although these prior studies did not observe differences between VKA and DOACs, we found that VKA users had a higher risk of thromboembolism, which supported several previous studies demonstrating that DOAC therapy is more effective and even as safe as VKA therapy [47, 48].

DOACs have recently demonstrated to be superior in comparison to VKAs among patients with different conditions. For example, DOACs was associated with lower long-term all-cause mortality than VKAs in AF patients who were successfully discharged after transcatheter aortic valve replacement [49]. In addition, in patients with left ventricular thrombi, there is a significant reduction in stroke with the use of DOACs, without an increase in bleeding [50], and among diabetic AF patients, DOACs are associated with a lower risk of thromboembolism, major bleeding, and major adverse limb events than VKAs [51].

Nonetheless, the published data for DOACs in the field of COVID-19 is conflicting. In the context of hospitalization, many of the COVID-19 patients with prior DOAC therapy will be switched to heparins because they are expected to receive medications interacting with DOACs, or have coagulation system and homeostasis disorders [52, 53]. For this reason, it is necessary to act before hospitalization in these especially vulnerable patients. Since our results show that within 30 days of COVID-19 diagnosis there is an increased risk of complications in patients on prior VKA, whenever possible, a change to a DOAC should be considered. This may not only reduce the risk of adverse events in the case of COVID-19, but also the need for monitoring and therefore the number of visits to health care centers and the social contact that this implies, which can contribute to a lower risk of infection [54]. Some societies consider that DOAC could be maintained even during hospitalization, based on clinical conditions and assessment for drug–drug interactions [21, 55, 56].

However, there are some indications such as antiphospholipid syndrome or prosthetic heart valves for which DOACs are not approved. In such patients, a switch from VKA to DOACs is not possible, and therefore other interventions are required to achieve and maintain the highest quality of anticoagulation in order to reduce the risk of worse clinical outcomes when on VKAs. These include the identification and modification of causes of poor anticoagulation control with VKAs (eg. potential pharmacologic interactions), routine assessment of adherence to treatment, patient-centred education/counselling and educational programs for healthcare professionals, the use of multidose drug dispensing systems, self-management of VKA therapy, specialized anticoagulation clinics to improve adherence and a more careful follow-up [57, 58]. Due to the COVID-19 pandemic, telemedicine visits and telehealth programs may help to achieve these objectives while minimize exposure risks for both patients and healthcare professionals [59].

### Limitations

There are some limitations that need to be acknowledged. First, the data were collected from the HCO EMRs and some health conditions may be underreported. Recording of ICD codes in EMR may vary by factors including age, comorbidities, severity of illness, length of in-hospital stay, and in-hospital mortality. Further residual confounding may include lifestyle factors such as alcohol consumption and physical activity, which were not available.

Propensity scores are a method used to balance covariates, but in observational studies propensity scores are estimated and therefore there is no certainty that the propensity score was 100% accurate. We also could not determine if there was any impact of attending different HCOs because of data privacy restrictions. We examined all deaths of the included patients captured within the TriNetX network; however, deaths outside of the participating HCOs are not well captured. It should also be noted that we had not access to time in therapeutic range of International Normalized Ratio (INR) for VKA-treated patients, so there are uncertainties about the quality of anticoagulation therapy in these patients. Finally, our main objective was to investigate the association of prior VKA or DOAC therapy with short-term prognosis after outpatient COVID-19 diagnosis. For this reason we did not take into account the anticoagulation therapy once patients were diagnosed of COVID-19, since our interest was on the previous use of VKAs or DOACs. For the same reason, we have not analyzed the potential role of other pharmacological therapies such as antidiabetics agents or insulin, although we recognize influence of such therapies on outcomes.

## Conclusion

In COVID-19 outpatients with cardiometabolic diseases, prior use of DOAC therapy compared to VKA therapy at the time of COVID-19 diagnosis might reduce the risk of composite arterial or venous thrombotic outcomes, without increasing the risk of bleeding.

## Supplementary Information

**Additional file 1: Table S1.** ICD-10-CM codes and LOINCs for the identification of COVID-19. **Table S2.** Clinical outcomes and ICD-10-CM codes.

## Data Availability

The data, analytic methods, and study materials will not be made available to other researchers for purposes of reproducing the results or replicating the procedure because some materials are used for other unpublished projects.

## Abbreviations

AF: Atrial fibrillation
CDC: Centres for Disease Control and Prevention
CI: Confidence interval
COVID-19: Coronavirus Disease 2019
DOAC: Direct-acting oral anticoagulant
EMR: Electronic medical record
HCO: Healthcare organisation
HR: Hazard ratio
ICH: Intracranial haemorrhage
ICU: Intensive care unit
INR: International normalized ratio
MV: Mechanical ventilation
OAC: Oral anticoagulant
PSM: Propensity score matching
SARS-CoV-2: Severe Acute Respiratory Syndrome Coronavirus 2
SD: Standard deviation
SE: Systemic embolism
SMD: Standardised mean difference
TIA: Transient ischemic attack
VKA: Vitamin K antagonist
VTE: Venous thromboembolism
