# Supplementary material for: Efficacy and safety of direct-acting oral anticoagulants compared to vitamin K antagonists in COVID-19 outpatients with cardiometabolic diseases
Source: Cardiovasc Diabetol. 2021 Sep 4;20:176. doi: 10.1186/s12933-021-01368-6 (PMC8417638; doi:10.1186/s12933-021-01368-6)
Supplement: Supplementary file 1 — Additional file 1: Table S1. ICD-10-CM codes and LOINCs for the identification of COVID-19. Table S2. Clinical outcomes and ICD-10-CM codes. [file 12933_2021_1368_MOESM1_ESM.docx]

**Additional file**

**Table S1.** ICD-10-CM codes and LOINCs for the identification of COVID-19.

| - U07.2 Covid-19, virus not identified (who) - U07.1 Covid-19 - B97.29 Other coronavirus as the cause of diseases classified elsewhere - B34.2 Coronavirus infection, unspecified - 94564-2 Sars coronavirus 2 igm ab [presence] in serum or plasma by immunoassay [Positive] - 94563-4 Sars coronavirus 2 igg ab [presence] in serum or plasma by immunoassay [Positive] - 94559-2 Sars coronavirus 2 orf1ab region [presence] in respiratory specimen by naa with probe detection [Positive] - 94558-4 Sars coronavirus 2 ag [presence] in respiratory specimen by rapid immunoassay [Positive] - 94534-5 Sars-cov-2 (covid19) rdrp gene [presence] in respiratory specimen by naa with probe detection [Positive] - 94533-7 Sars-cov-2 (covid19) n gene [presence] in respiratory specimen by naa with probe detection [Positive] - 94508-9 Sars coronavirus 2 igm ab [presence] in serum, plasma or blood by rapid immunoassay [Positive] - 94507-1 Sars coronavirus 2 igg ab [presence] in serum, plasma or blood by rapid immunoassay [Positive] - 94506-3 Sars-cov-2 (covid19) igm ab [units/volume] in serum or plasma by immunoassay [≥ 0.1 units] - 94505-5 Sars-cov-2 (covid19) igg ab [units/volume] in serum or plasma by immunoassay [≥ 0.1 units] - 94502-2 Sars-related coronavirus rna [presence] in respiratory specimen by naa with probe detection [Positive] - 94500-6 Sars-cov-2 (covid19) rna [presence] in respiratory specimen by naa with probe detection [Positive] - 94316-7 Sars-cov-2 (covid19) n gene [presence] in unspecified specimen by naa with probe detection [Positive] - 94315-9 Sars-cov-2 (covid19) e gene [presence] in unspecified specimen by naa with probe detection [Positive] - 94309-2 Sars-cov-2 (covid19) rna [presence] in unspecified specimen by naa with probe detection [Positive] - 9089 Sars coronavirus 2 igg igm ab [presence] in serum or plasma [Positive] - 9088 Sars coronavirus 2 and related rna [presence][Positive] |
| --- |

**Table S2.** Clinical outcomes and ICD-10-CM codes.

| **Outcomes** | **ICD-10-CM codes.** |
| --- | --- |
| **All-cause mortality** | Deceased |
| **Intensive care unit admission/mechanical ventilation necessity** | The composite of any of the following:   - 5A09 [Physiological Systems / Assistance / Respiratory] - 1013729 [Critical Care Services] - 1013661 [New or Established Patient Initial Hospital Inpatient Care Services] |
| **ICH/gastrointestinal bleeding** | The composite of any of the following:   - I60 [nontraumatic subarachnoid hemorrhage] - I61 [nontraumatic intracerebral hemorrhage] - I62 [other and unspecified nontraumatic intracranial hemorrhage] - K92.0 [hematemesis] - K92.1 [melena] - K92.2 [gastrointestinal hemorrhage, unspecified] |
| **Composite of any arterial or venous thrombotic event** | The composite of any of the following:   - G45 [transient cerebral ischemic attacks and related syndromes] - I63 [cerebral infarction] - I67.82 [cerebral ischemia] - I74 [arterial embolism and thrombosis] - I26 [pulmonary embolism] - I81 [portal vein thrombosis] - I82 [other venous embolism and thrombosis] - I21 [acute myocardial infarction] - I22 [subsequent ST elevation and non-ST elevation myocardial infarction] |
| **Hospitalization** | The composite of any of the following:   - 1013659 [Hospital inpatient services] - 1013699 [Initial inpatient consultation services] - 1013729 [Critical care services] - Visit: inpatient non-acute - Visit: short stay - Visit: inpatient acute - Visit: inpatient encounter |
| **Myocardial infarction** | The composite of any of the following:   - I21 [acute myocardial infarction] - I22 [subsequent ST elevation and non-ST elevation myocardial infarction] |
| **Venous thromboembolism** | The composite of any of the following:   - I26 [pulmonary embolism] - I81 [portal vein thrombosis] - I82 [other venous embolism and thrombosis] |
| **Ischemic stroke/TIA/SE** | The composite of any of the following:   - G45 [transient cerebral ischemic attacks and related syndromes] - I63 [cerebral infarction] - I67.82 [cerebral ischemia] - I74 [arterial embolism and thrombosis] |
| **All bleeding** | The composite of any of the following:   - Hemorrhage, not elsewhere classified - K92.0 [hematemesis] - K92.1 [melena] - K92.2 [gastrointestinal hemorrhage, unspecified] - R04.0 [Epistaxis] - R04.1 [Hemorrhage from throat] - R04.2 [Hemoptysis] - K62.5 [Hemorrhage of anus and rectum] - D68.3 [Hemorrhagic disorder due to circulating anticoagulants] - I60 [nontraumatic subarachnoid hemorrhage] - I61 [nontraumatic intracerebral hemorrhage] - I62 [other and unspecified nontraumatic intracranial hemorrhage] |
